# A Novel Surgical Approach for the Management of Cloacal Exstrophy with a Giant Omphalocele

**DOI:** 10.1055/s-0041-1728719

**Published:** 2021-05-18

**Authors:** Caitlin A. Smith, Jeffrey R. Avansino, Paul Merguerian, Victoria Lane, Marc Levitt

**Affiliations:** 1 Department of General and Thoracic Surgery, Seattle Children's Hospital, University of Washington School of Medicine, Seattle, Washington, United States; 2 Department of Urology, Seattle Children's Hospital, University of Washington School of Medicine, Seattle, Washington, United States; 3 Department of Surgery, The Great North Children's Hospital, Royal Victoria Infirmary, Newcastle, United Kingdom; 4 Division of Colorectal and Pelvic Reconstructive Surgery, Children's National Hospital, Washington, District of Columbia, United States

**Keywords:** cloacal exstrophy, bladder exstrophy, anorectal malformation

## Abstract

Cloacal exstrophy is a rare malformation that presents as a lower midline abdominal wall defect which affects the gastrointestinal and genitourinary systems. The components of cloacal exstrophy characteristically include omphalocele, exstrophy of perineal structures, and imperforate anus. Most of these patients also have renal anomalies such as pelvic kidney, fused kidneys, or solitary kidneys. This congenital condition can also be associated with spinal issues, such as spinal dysraphism. When combined with spinal defects, it is referred to as the omphalocele, exstrophy, imperforate anus, and spinal defects (OEIS) complex, and is one of the most challenging surgical conditions to manage. Here, we present a unique case of a low-birth-weight patient with OEIS and a liver containing giant omphalocele and the novel surgical technique used to manage her cloacal exstrophy whereby the cecal plate was not separated from the bladder halves, but rather left for an autoaugment, and the ileum was connected to the hindgut.

## Introduction

Cloacal exstrophy is a rare congenital condition when abnormal closure of the abdominal wall results in an open bladder divided by the cecal plate in a lower midline defect. This congenital disorder is the most severe of the conditions included in the exstrophy-epispadias complex (EEC). Although first described in 1709, the first surgical repair was not attempted until 1900. 1 Mortality was high in the initial cases with malnutrition and sepsis being the primary postoperative complications. However, since about the 1970s, mortality has declined to ∼10% and remained at this level until today. 2

The true incidence of cloacal exstrophy is difficult to know, but it is estimated at ∼1:200,000 to 1:400,000 live births and may be higher if stillbirths are included. 3 The etiology of cloacal exstrophy is generally unknown as there are no consistent genetic or environmental factors that have been confirmed as causative. There are suggestive relationships between EEC and in vitro fertilization 4 as well as the use of clomiphene citrate (an estrogen receptor inhibitor); however, these relationships have only been identified in small studies that had other confounding factors. 5

Surgical repair of cloacal exstrophy often requires an individualized approach in each case. While the individual details of each surgical intervention may vary, the goals of surgery traditionally have been the same: to separate the cecum from the bladder plates and create a stoma, to close the bladder primarily or in a delayed form, and closure of the abdominal wall.

In this specific case, the patient was premature, born at 32 weeks, and at a low birth weight, with a giant liver containing omphalocele, as defined as an abdominal wall defect more than 5 cm and herniation of more than 50% of the liver. 6 Due to these factors, the typical surgical strategies could not be applied. Here, we present a unique surgical approach to the patient with cloacal exstrophy and giant omphalocele that could be applicable to other cloacal exstrophy patients with similar anatomy.

## Case History

**Fig. 1:**
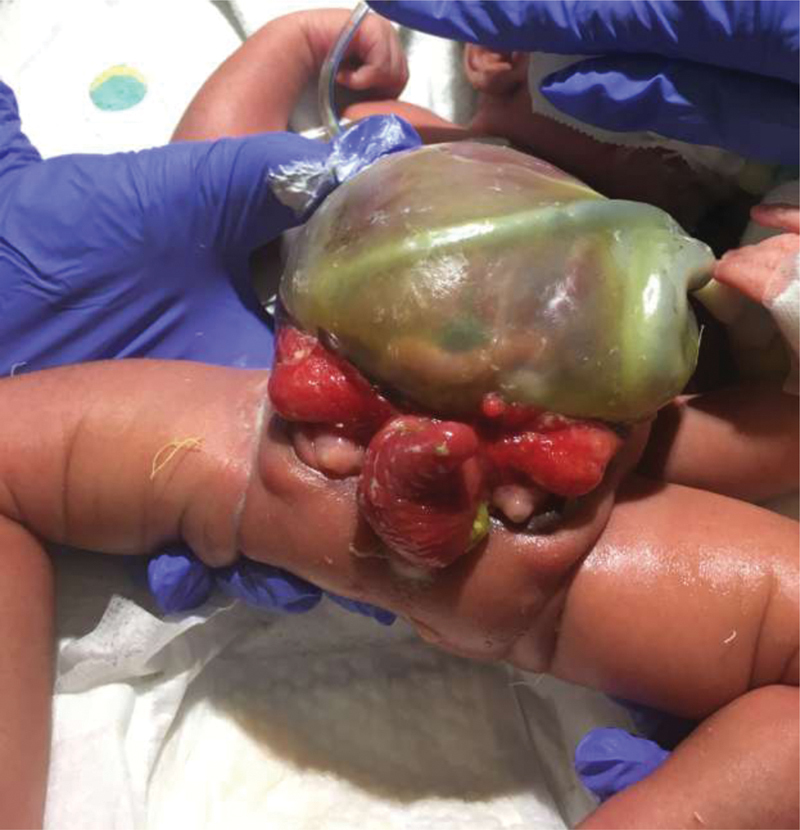
Day of life 1: Inferior aspect with bladder plates and intussuscepted ileum at cecal plate and large omphalocele.

**Fig. 2:**
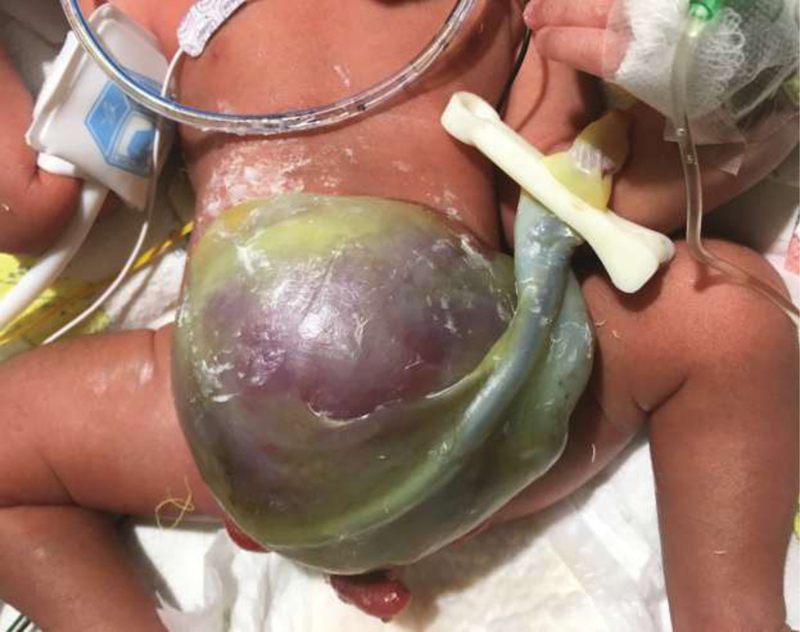
Day of life 1: Large liver containing omphalocele.

The patient is a 32-week premature infant with a prenatal diagnosis of cloacal exstrophy. She was born via cesarean section due to fetal decelerations and concern for fetal distress. She was twin B of a twin pregnancy, and her sister was healthy. At delivery, her Apgar scores were 2 and 8 at 1 and 5 minutes, respectively. Her birth weight was 1.7 kg. She was intubated shortly after delivery for respiratory distress and once stabilized, she was transferred to a quaternary care facility for further surgical management. On physical examination, she had a giant liver containing omphalocele that was not amenable to the typical surgical strategy of early closure and conversion to bladder exstrophy ( Figs. 1 and 2 ). Due to her small size and giant omphalocele, topical silver sulfadiazine paint was applied to the omphalocele and hydrogel dressings to the exstrophy portion as a nonoperative management strategy, so she could grow prior to surgical intervention. During this period, she was fed via a nasogastric tube; however, she did not tolerate full feeds due to both dumping symptoms and frequent requirement of dressing changes. Genetic testing was performed, and her karyotype was 46,XX. She was transitioned to trickle feeds until she underwent operative intervention at 3 months old and ∼3.5 kg. She successfully drained stool from the cecal plate and urine from the bladder halves. The bladder plates and bowel were managed with hydrogel dressing with a double diaper throughout this period. This did require intensive nursing care and changing every 6 to 8 hours. The omphalocele sac had also completely epithelialized at the time of surgical intervention.

Due to the large nature of the omphalocele, abdominal closure at the time of the initial operation was not possible. The primary goal of this operation was to be able to feed the child enterally. As the abdominal wall could not be closed due to the large omphalocele, a novel approach was needed to bridge the space between the bladder halves. This was performed with a terminal ileum bowel flap and leaving the cecal plate in situ which essentially autoaugmented the bladder. The operative technique is described later.

### Operative Technique

The operation was initiated with a transverse laparotomy incision at the inferior edge of the epithelialized omphalocele sac. Once the intra-abdominal contents were reached, the cecal plate was examined. The very distal ileum was intussuscepted externally and was reduced. The terminal ileum was identified and eviscerated from the abdomen, and was followed to its junction with the cecal plate. The terminal ileum was divided from the cecal plate, leaving the cecal plate and some ileum attached between the bladder halves.

**Fig. 3:**
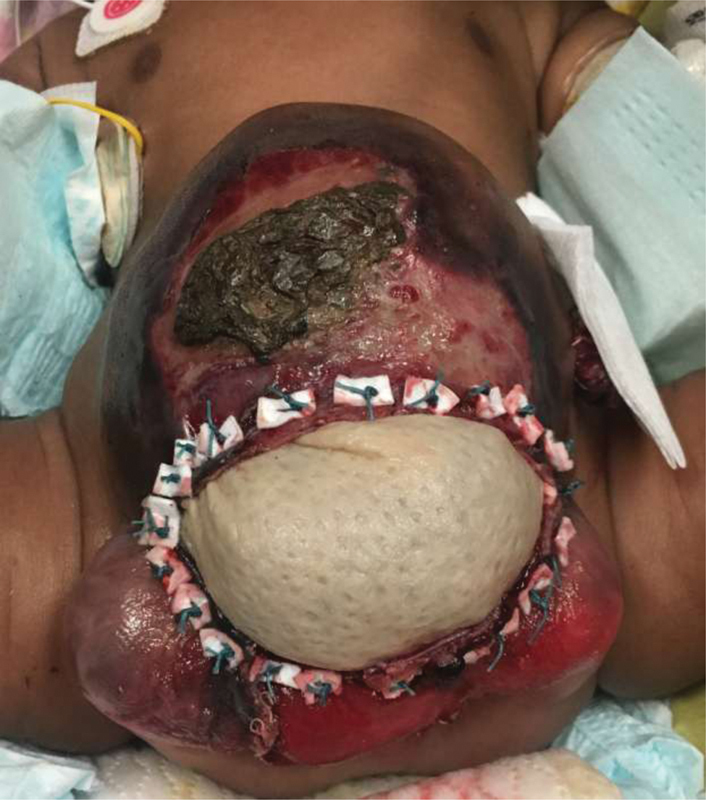
Three months old: Postrepair with bowel patch between bladder plates and alloderm closure of omphalocele. Ostomy is in the left upper quadrant, under the gauze.

The hindgut was identified and mobilized. The hindgut was separated from the surrounding structures. As the bladder halves could not be brought together in the presence of the large omphalocele, a segment of ileum and some of the cecum were opened along the antimesenteric border to function as a patch between the bladder plates which bridged this space. The proximal hindgut was sutured in an end-to-end fashion to the divided terminal ileum, and was brought out as an ostomy in the left lateral abdomen, outside of the omphalocele sac. Closure of the omphalocele sac was performed with an alloderm patch with pledgeted U-sutures as the liver became swollen during the surgery, and primary closure was not possible due to concern for increased tension and risk of compartment syndrome ( Fig. 3 ).

**Fig. 4:**
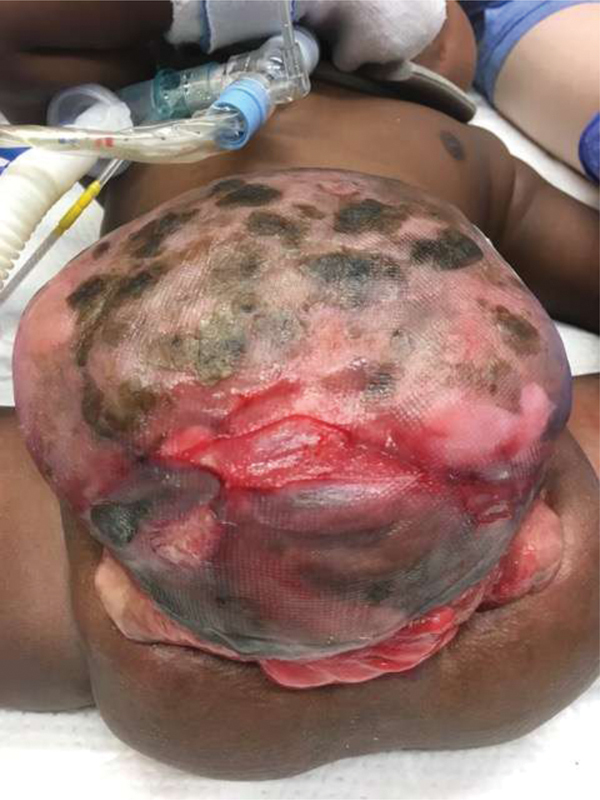
Twelve months old: After alloderm patch has re-epithelialized. Bladder plates and bowel on inferior aspect of omphalocele.

Postoperatively, the patient did well. The abdominal wound healed, the alloderm epithelialized entirely, and enteral feeds were achieved without symptoms or evidence of dumping. As of 12 months old, her omphalocele and bladder exstrophy closure had still not occurred due to the relative size of omphalocele ( Fig. 4 ).

## Discussion

Cloacal exstrophy represents a significant surgical challenge that requires a multidisciplinary surgical team approach and traditionally has several primary goals. First, to separate the cecum from the bladder plates, tubularize it, and mobilize the hindgut as an end stoma. This maintains bowel length and exposes the hindgut to the fecal stream, so it can lengthen. 7 The urinary system is initially allowed to drain freely usually from the two bladder halves to preserve renal function. Additional goals include closing the abdominal wall and closure of the bladder, either primarily or in a delayed fashion. Sometimes, these goals may be achieved in a single-stage procedure, but more commonly, the procedures are not performed concurrently. The traditional description of the newborn operation is to separate the cecal plate from the center of the two bladder halves and to close the omphalocele, converting the patient effectively to a pure bladder exstrophy. Subsequent bladder exstrophy closure is performed in the future, when the patient is at an appropriate age. Pelvic osteotomies are also needed at this time.

Ultimately, further definitive surgical reconstruction is typically performed when the patient is older to achieve social continence for urine and stool, including usually bladder augmentation, bladder neck closure or reconstruction, vaginal reconstruction if appropriate, Malone appendicostomy, and Mitrofanoff. While historically it was thought that patients with cloacal exstrophy had a short and useless colon, with care for colon preservation at the initial operation, these children can often undergo a pull through procedure at and achieve social continence with bowel management. This is ideally performed at the same time as the urologic reconstruction. 7 8

In contrast to the above-mentioned surgical strategy, in the case presented, a unique surgical approach was performed to achieve enteral feeding, when omphalocele closure and conversion to bladder exstrophy could not be performed based on anatomic factors specific to this patient. An ileal bowel/cecal plate flap was used to bridge the gap between the bladder halves.

**Fig. 5:**
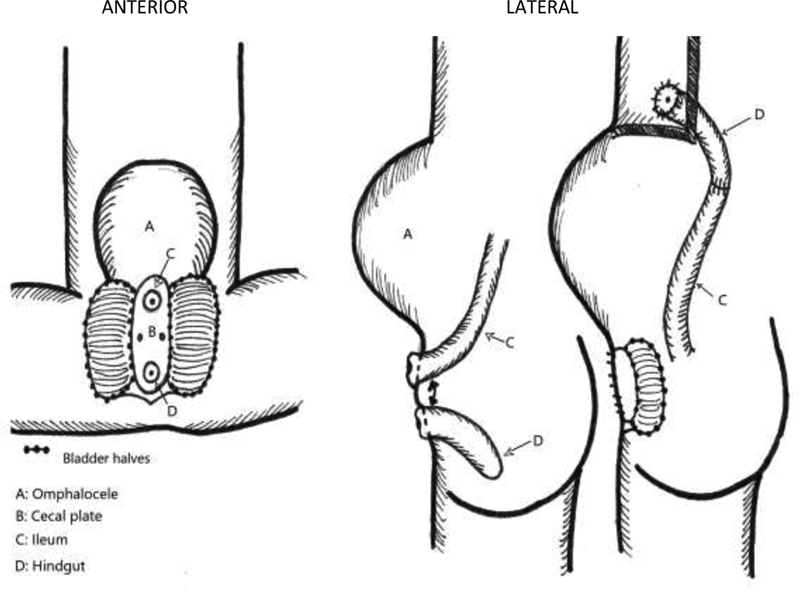
Descriptive diagram of surgical intervention depicting anastomosis of ileum to hindgut once separated from the cecal plate. Bladder halves and cecal plate were left in situ due to the large omphalocele.

In the past, concerns have existed for development of acidemia caused by urine absorption by intestinal mucosa; however, this did not occur in this patient and is likely not a long-term concern in practice. We already know this is rarely a concern in bladder-augmented patients. This leads us to consider whether the concept of always separating the cecal plate from between the bladder halves is even necessary. It could be recommended that in some cases, the surgeon can leave the cecal plate intact leaving it and the very distal ileum attached to the hindgut. This sets up a more size matched and easier to perform anastomosis between distal ileum and proximal hindgut, and tubularization of the cecal plate can be avoided ( Fig. 5 ).

Preserving colonic length is vital for achieving appropriate stool quality for future pull through and achieving social continence. 7 8 It is unclear if the cecal plate contributes that much to this goal, and could potentially be a source of fecal stasis and bacterial overgrowth in the future. 9 An additional benefit of leaving the cecal plate or ileum in situ includes improving the ease of the initial newborn operation. Furthermore, as the majority of these patients will require bladder augmentation with bowel and an antegrade catheterizable channel, the bowel left in situ between the bladder plates can be considered an autoaugmentation of the bladder. We believe that this newborn intervention will allow for a larger bladder capacity at the time of bladder closure thus reducing the risk to the upper urinary tracts and may preclude the future need for an augmentation.

## Conclusion

It is clear that these patients can have varying anatomic considerations, and an individualized surgical approach should be developed at the initial surgery to provide the best possibility of long-term success. In summary, this case illustrates the following learning points:

1. There is no urgency to the newborn repair: the giant omphalocele can be painted, the urine can drain via the extrophied bladders, and the stool can exit via the cecal plate.
2. The cecal plate and distal ileum can be left between the bladder halves—this does not lead to acidosis as formerly thought and potentially may serve as autoaugmentation of the bladder. This could add benefits of assisting with the future reconstruction and allowing for a larger bladder at initial closure preserving kidney function.
3. Not tubulalizing the cecal plate can avoid it being a nidus for future stasis and bacterial overgrowth.
4. The ileum to hindgut anastomosis is nicely size matched and technically easier than tubularizing the cecal plate.
5. Long-term follow-up is needed to confirm the benefits of this surgical strategy.

## Notes

New Insights and the Importance for the Pediatric Surgeon This case report presents a new take on a complex surgical problem and management of cloacal exstrophy. The authors believe this may represent a more straightforward surgical approach, with the added benefit of autoaugmentation of the subsequent bladder extrophy.
